# IL-23 p19 Knockout Mice Exhibit Minimal Defects in Responses to Primary and Secondary Infection with *Francisella tularensis* LVS

**DOI:** 10.1371/journal.pone.0109898

**Published:** 2014-10-08

**Authors:** Sherry L. Kurtz, Alicia Y. Chou, Klara Kubelkova, Daniel J. Cua, Karen L. Elkins

**Affiliations:** 1 Laboratory of Mycobacterial Diseases and Cellular Immunology, Division of Bacterial, Parasitic and Allergenic Products, Center for Biologics Evaluation and Research, U.S. Food and Drug Administration, Rockville, Maryland, United States of America; 2 Pathway Biology, Merck Research Laboratories, Palo Alto, CA, United States of America; Midwestern University, United States of America

## Abstract

Our laboratory’s investigations into mechanisms of protective immunity against *Francisella tularensis* Live Vaccine Strain (LVS) have uncovered mediators important in host defense against primary infection, as well as those correlated with successful vaccination. One such potential correlate was IL-12p40, a pleiotropic cytokine that promotes Th1 T cell function as part of IL-12p70. LVS-infected IL-12p40 deficient knockout (KO) mice maintain a chronic infection, but IL-12p35 KO mice clear LVS infection; thus the role that IL-12p40 plays in immunity to LVS is independent of the IL-12p70 heterodimer. IL-12p40 can also partner with IL-23p19 to create the heterodimeric cytokine IL-23. Here, we directly tested the role of IL-23 in LVS resistance, and found IL-23 to be largely dispensable for immunity to LVS following intradermal or intranasal infection. IL-23p19 KO splenocytes were fully competent in controlling intramacrophage LVS replication in an *in vitro* overlay assay. Further, antibody responses in IL-23p19 KO mice were similar to those of normal wild type mice after LVS infection. IL-23p19 KO mice or normal wild type mice that survived primary LVS infection survived maximal doses of LVS secondary challenge. Thus p40 has a novel role in clearance of LVS infection that is unrelated to either IL-12 or IL-23.

## Introduction

*Francisella tularensis* is an intracellular bacterium that causes tularemia in humans, an infection which may present in different forms depending on the route of inoculation [1]. A live attenuated vaccine strain (denoted LVS) was developed using *F. tularensis* type B *holarctica* [2], [3]. While this vaccine is not licensed for use in humans, it is an excellent tool for studying both primary and secondary immunity to *F. tularensis* in various animal model systems, including mice, rats, guinea pigs, and rabbits [4], [5].

The murine model has been used to study primary and adaptive immunity to virulent strains of *F. tularensis*, as well as to LVS. Mice survive primary infection when LVS is given via the intradermal (ID) or intranasal (IN) routes, with an LD_50_ of ∼10^6^ or 10^3^ for each route, respectively [4]. Once vaccinated by primary infection, mice survive high doses of secondary challenge by either IN delivery or by intraperitoneal (IP) injection, a route by which the LD_50_ is <10 CFU in naïve mice.

The LVS infection model has been used to demonstrate the importance of several immune effectors in resistance to LVS, such as CD4^+^ T cells, CD8^+^ T cells, and CD4^−^CD8^−^ double negative T cells [4], [6]. A number of T cell-derived cytokines have been shown to be essential for full immunity to LVS, including IFN-γ and TNF-α. IFN-γ and TNF-α production are increased by IL-12, a heterodimeric cytokine comprised of covalently linked p40 and p35 subunits that form the 70 kDa mature protein [7]. Of the two subunits, the expression of p35 is the most tightly regulated. The p40 subunit is produced in excess and can itself form a biologically active homodimer, or can exist as a free monomer. The IL-12 receptor (IL-12R) complex is also a heterodimer, comprised of IL-12Rβ1 and IL-12Rβ2 subunits. IL-12 promotes T cell development and natural killer (NK) cell function, and participates in a feedback loop to regulate antigen presenting cell (APC) function. In the context of the host response to microbial pathogens, IL-12 stimulates IFN-γ production and Th1 responses, which in turn control infection via T cell activities and direct stimulation of bactericidal actions of macrophages. Presumably due to its role in the Th1 response, IL-12 is important for host resistance to many intracellular pathogens, including *Cryptococcus neoformans*, *Toxoplasma gondii*, *Salmonella enteritidis*, and *Mycobacterium tuberculosis* [8]–[11].

IL-12 also plays a role in protection against *F. tularensis* LVS infection, albeit a complicated one. IL-12 is rapidly induced in the skin of mice following intradermal LVS infection [12]. However, IL-12p35 knockout (KO) mice exhibit mortality rates similar to those of wild type (WT) animals when infected with LVS intradermally (ID), except when given a very high dose (10^7^) [13]. IL-12p35 KO mice had delayed bacterial clearance compared to WT, but nonetheless cleared the infection by day 24. IL-12p40 KO mice also had initial mortality rates similar to WT mice for various doses of ID LVS infection; further, immune splenocytes from both LVS-vaccinated IL-12p35 and IL-12p40 knockout mice controlled intracellular LVS growth in an *in vitro* assay, demonstrating that these mice developed successful T cell responses in the absence of IL-12p40 or IL-12p35. However, while the bacterial burdens in IL-12p40 KO mice infected ID with LVS decreased until day 7–14, thereafter the bacterial burdens remained static until at least day 78 and beyond. Thus IL-12p40 KO mice maintain a chronic LVS infection apparently for the life of the mouse. IL-12p40 KO mice and IL-12p35 KO mice are both more susceptible to IN LVS infection and have reduced LD_50_s compared to WT mice [14]; Elkins *et al.*, unpublished data]; but IL-12p40 KO mice are also unable to clear LVS after low dose IN infection. Thus, given the disparity of phenotypes between the IL-12p40 and IL-12p35 KO mice during LVS infection, there appears to be role for IL-12p40 in controlling LVS infection that is independent of its action in the IL-12p70 heterodimer.

IL-23 is a heterodimeric cytokine related to IL-12 through shared subunits in both the mature cytokine and receptor [15], [16]. IL-23 is comprised of the IL-12p40 subunit and an IL-23p19 subunit whose structure is closely related to IL-12p35. The receptor for IL-23, IL-23R, is comprised of IL-12Rβ1 and IL-23R subunits. IL-23 itself is mainly produced by cells of the innate immune system including dendritic cells (DCs) and macrophages, upon stimulation of certain toll like receptors (TLRs), predominantly TLR2, and to a lesser extent via TLR4 [17]. IL-23 also has a role in the innate and adaptive immune responses to several bacterial pathogens, including the control of primary infection by *L. monocytogenes*, *K. pneumonia,* and *M. tuberculosis* [18]–[20]. In the context of adaptive immunity, successful immunization against *S. aureus* and *M. tuberculosis* is partially dependent on IL-17, IL-23, and the Th17-IL-23 pathway [21]–[23].

Given the disparity in the phenotypes between IL-12p40 KO mice and IL-12p35 KO mice during LVS infection, here we examined the role of p19 as a component of IL-23 during systemic and respiratory LVS infection, using IL-23p19 KO mice [24], [25].

## Materials and Methods

### Mice

Male and female C57BL/6J mice 6–8 weeks of age were purchased from Jackson Laboratories (Bar Harbor, Maine), and acclimated for at least a week before use. C57BL/6 IL-23p19 KO mice were obtained from two sources [24], [25]. Here, KO mice obtained from Merck are designated as p19M KO and those from Genentech as p19G KO. Each version of the IL-12p19 knockout mouse was created with a unique deletion construct; both versions result in large deletions in the IL-23p19 gene. To the best of our knowledge, there have been no reported phenotypic differences between the two knockout strains. All mice were housed in microisolator cages, and were given autoclaved food and water *ad libitum*. Animal studies using LVS were performed under protocols approved by the Animal Care and Use Committee (ACUC) of CBER/FDA. Within each experiment, all animals were age- and sex-matched.

### Bacteria and growth conditions

*F. tularensis* LVS (American Type Culture Collection 29684) was grown in modified Mueller-Hinton (MH) broth (Difco Laboratories, Detroit Michigan) to mid-logarithmic phase as previously described [26], then frozen in 0.5 ml aliquots at −70°C until use. Using adult male BALB/cByJ mice, a sample from each batch of bacterial stock was subjected to quality control experiments to determine the number of colony forming units (CFU), to determine the proportion of dead bacteria (Live/Dead BacLight Bacterial Viability kit, Invitrogen), to confirm typical colony morphologies, and to confirm the expected IP and ID LD_50_s and expected time to death [27].

### Bacterial infections

C57BL/6J and the indicated KO mice were infected ID with the indicated doses, delivered in 0.1 ml of sterile phosphate buffered saline (PBS) (Lonza, Walkersville, MD) containing <0.01 ng of endotoxin/ml, or given IP in 0.5 ml PBS. Mice were also infected by the IN route. Before IN infection, mice were anesthetized with 0.1 ml of a cocktail of Ketaject ketamine HCl (1.5 mg/0.1 ml) (Phoenix Pharmaceuticals, St. Joseph, MO) and AnaSed (0.3 mg/0.1 ml) (Lloyd Laboratories, Shenandoah, IO) diluted in sterile PBS and given IP. Mice were infected IN with the indicated doses of LVS delivered in a 20 µl volume to a single nostril. Each LVS infection dose was plated on MH plates to assess the actual number of CFU delivered, and the colonies enumerated after 2–3 days incubation at 37°C/5% CO_2_. After infection, mice were monitored and were euthanized when clearly moribund, according to established protocols.

### Assessment of bacterial organ burdens and tissue pathology

Bacterial burdens in the organs were determined at the indicated time points after infection. Mice were euthanized, and organs removed aseptically and transferred to sterile homogenizer bags containing 5 ml of sterile PBS/organ. Organs were disrupted using a Stomacher (Seward, England), and the homogenates serially diluted and plated for colony enumeration. Organ homogenates were also frozen and stored at −70°C to be used for cytokine analyses.

### Characterization of antibody response

Titers of specific anti-LVS serum antibodies were determined by ELISA as described previously [28]. Briefly, Immulon 1 plates were coated with live LVS, washed, and blocked with 10% calf serum. Serum samples were added using two-fold serial dilutions. In each assay, sera from naïve mice was used as a negative control, and monoclonal anti-LVS IgM and sera from LVS-hyperimmune mice were used as positive controls. Horseradish-peroxidase labeled antibodies (anti-IgM and anti-IgG that detects IgG_1_, IgG_2a_, IgG_2b_, and IgG_3_) (Southern Biotech, Birmingham, AL) were added, and ABTS peroxidase substrate (Kirkegaard and Perry Laboratories, Gaithersburg, MD) was used for color development. The end point titer was defined as the reciprocal of the lowest dilution of serum that gave an optical density at 405 nm greater than the optical density at 405 nm of the matched dilution of normal pre-bleed mouse serum plus three standard deviations added to the OD value, and also greater than 0.025 [28]. When the sera were collected for each time point, groups of five mice were bled individually, but equal volumes of their sera were pooled for testing by ELISA.

### Preparation of splenocytes

Spleens were aseptically removed and transferred to a sterile petri dish with PBS and 2% fetal bovine serum (FBS) (Hyclone, Logan, CT). The plunger of a 3 ml sterile syringe was used to homogenize spleens, and the homogenate was passed through 100 µM and 40 µM cell strainers successively to achieve a single cell suspension. Red blood cells were lysed using ACK lysis buffer (Lonza, Walkersville, MD). Cells were washed in PBS/FBS; an aliquot was stained with trypan blue dye to determine cell viability, and cells enumerated using a hemocytometer.

### In vitro overlay assay

Co-cultures were performed in 24 or 48 well tissue culture plates as described previously [13], [29], [30]. Briefly, bone marrow-derived macrophages were differentiated by culture in complete DMEM (DMEM supplemented with 10% heat-inactivated FBS (HyClone, Logan, UT), 10% L-929-conditioned medium, 0.2 mM L-glutamine, 10 mM HEPES buffer, and 0.1 mM nonessential amino acids) in 24 well plates. Confluent adherent macrophage monolayers were infected for 2 hours with *F. tularensis* LVS at a multiplicity of infection (MOI) of 1∶20 (bacterium-to-BMMØ), washed, treated for 45–60 min with 50 µg/ml gentamicin, and washed extensively with antibiotic-free medium. Single-cell suspensions of splenic lymphocytes derived from vaccinated mice (5×10^6^/well, or as indicated) were added to LVS-infected macrophages. Supernatants from harvested cells were collected and stored at −70°C until analyzed for nitric oxide and cytokines as described below. Intracellular bacterial burdens in adherent infected macrophages were determined by lysing the macrophages with water, and plating the lysate as previously described [30].

### Cytokine and nitrite measurements

Organ homogenates and supernatants recovered from *in vitro* co-cultures were assayed using standard sandwich ELISAs, according to the manufacturer’s instructions (BD Pharmingen). The absorbance was read at 405 nm on a VersaMax tunable microplate reader with a reference wavelength of 630 nm (Molecular Devices, Sunnyvale, CA). Cytokine concentrations were determined by comparing unknown values to a standard curve prepared with recombinant protein at known concentrations (BD Pharmingen), using four-parameter fit regression in the SOFTmax Pro ELISA analysis software (Molecular Devices). Antibody pairs and standards were purchased from BD Pharmingen. Nitric oxide was estimated in culture supernatants by detecting nitrite using the Griess reaction [31]. Samples of supernatants were incubated with an equal volume of commercial Griess reagent (Life Technologies, Grand Island, NY) and absorbance was measured at 548 nm. Nitrite (NO_2_) was measured by comparison to serially diluted NaNO_2_ as a standard using four-parameter fit regression as described above.

### Statistical analyses

The statistical significance of differences within parameters was assessed using Student’s t test (SigmaPlot, Systat Software, Inc., San Jose, CA).

## Results

### Characterization of the role of IL-23p19 during primary LVS infection

**Figure 1:**
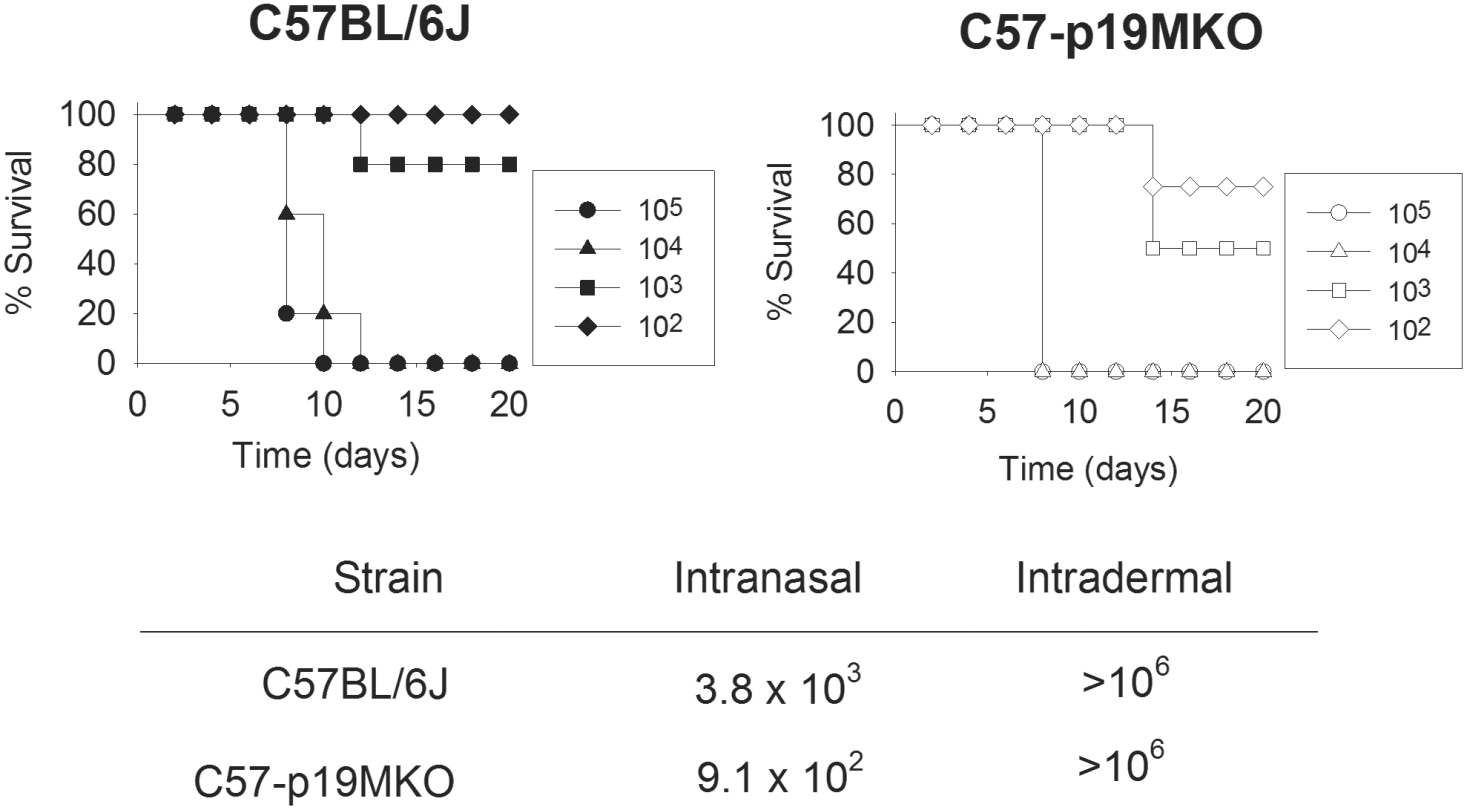
C57BL/6J and IL-23 deficient mice exhibit comparable survival patterns following IN or ID LVS infection. Groups of 5 to 10 WT mice and p19M KO mice were infected IN with LVS doses ranging from 10^1^–10^6^ CFU (doses 10^1^ and 10^6^ not shown for clarity). For ID infections, groups of 5 WT mice and p19M KO were infected ID with LVS doses ranging from 10^4^–10^6^. The calculated LD_50_ for IN and ID infections are shown (inset table). No mice from any ID-infected group succumbed to infection. Results shown are representative of three experiments of similar design and outcome. p19G KO mice were also included in some experiments with similar results (data not shown).

To determine the role that IL-23 plays in resistance to LVS, we employed IL-23 p19 deficient KO mice. In order to examine IL-23’s role as comprehensively as possible, we used two different strains of IL-23 p19 KO mice in our studies, one developed by Cua *et al.* [24], designated here as p19M KO, and a second strain developed by Ghilardi *et al.* [25], designated p19G KO. WT and p19M KO mice were infected with 10^4^–10^6^ LVS intradermally. No mice from any group succumbed to infection at any dose; therefore, the LD_50_ for either strain is >10^6^ (Fig. 1, inset chart). p19G KO mice were included in some experiments in more limited numbers, and also exhibited an LD_50_>10^6^ (data not shown). To evaluate susceptibility to IN LVS infection, WT and p19M KO mice were anesthetized and infected with 10^1^–10^6^ CFU LVS intranasally (Fig. 1; data not shown for 10^1^ and 10^6^). Both strains of mice exhibited similar survival patterns and thus similar LD_50_s, calculated to be ∼3.8×10^3^ for WT mice and ∼9.1×10^2^ for p19M KO mice (Fig. 1). Mice were weighed every three days during the course of the infection, and there were no obvious differences in weight loss or recovery of weight between the LVS-infected WT mice and p19M KO mice (data not shown). p19G KO mice were included in two experiments, with no differences observed in susceptibility between them and the WT or p19M KO mice (data not shown).

**Figure 2:**
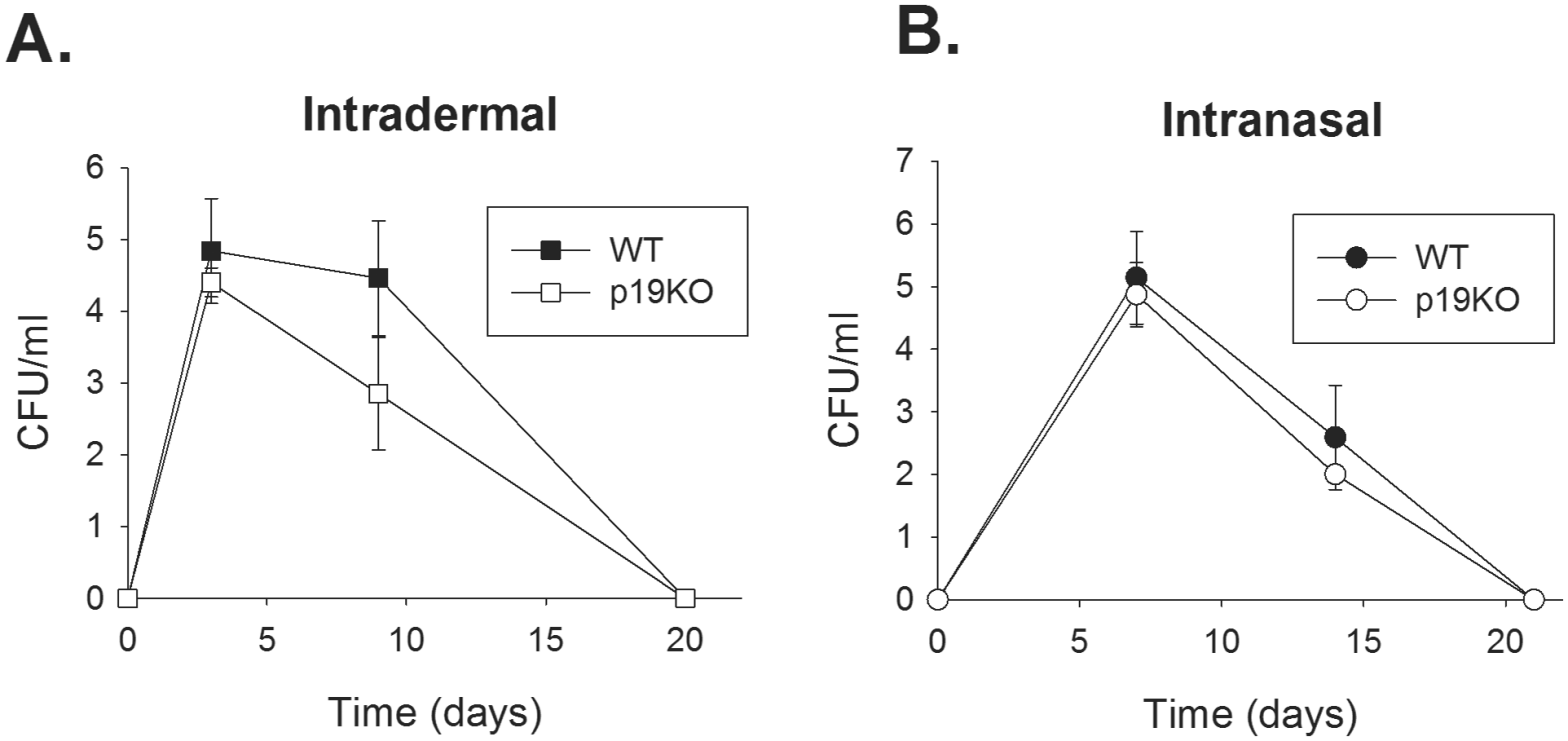
Bacterial burdens in tissues are comparable between infected WT mice and p19G KO mice infected with LVS ID and IN. Groups of 3 WT mice or p19G KO mice were infected either ID with 10^5^ LVS (A), or were anesthetized and infected with 2×10^3^ LVS IN (B). Mice were sacrificed at the indicated time points, and lungs, livers, and spleens plated to enumerate CFU. There were no significant differences in organ burdens between WT mice and p19G KO mice for any organ at any time point. Results shown are representative of two experiments of similar design and outcome. p19M KO mice were also included in one experiment with similar results (data not shown).

Although there were no obvious differences in LD_50_, we examined whether there were more subtle differences in the bacterial burdens in organs during IN or ID infection. WT mice and p19G KO mice were infected ID with 10^5^ LVS. Lungs, livers, and spleens were harvested at days 3, 9, and 20 after infection, and evaluated for total numbers of CFU per organ. There were no significant differences in organ burdens between the WT mice and p19G KO mice at any time point examined, in either spleens (Fig. 2A), livers, or lungs (data not shown). Similarly, WT mice and p19G KO mice were infected IN with 2×10^3^ LVS. Organs were harvested and evaluated for bacterial burdens at days 7, 14, or 21 after IN infection; there were no differences in bacterial burdens in either spleens (Fig. 2B), livers, or lungs (data not shown). p19M KO mice were included in one experiment for both ID and IN LVS infection, and their organ burden profiles were similar to those of LVS-infected WT mice and p19G KO mice (data not shown). Finally, for both infection routes, the levels of cytokines in the organ homogenates were measured at all timepoints. There were no differences in amounts of TNF-α, IFN-γ, MCP-1, IL-4, IL-12p40, or IL-12p70 in any organ between the LVS-infected WT mice and p19G KO mice (data not shown). Of particular note, there were also equivalent amounts of IL-17A in the organs of WT and p19G KO mice, including lung homogenates.

**Figure 3:**
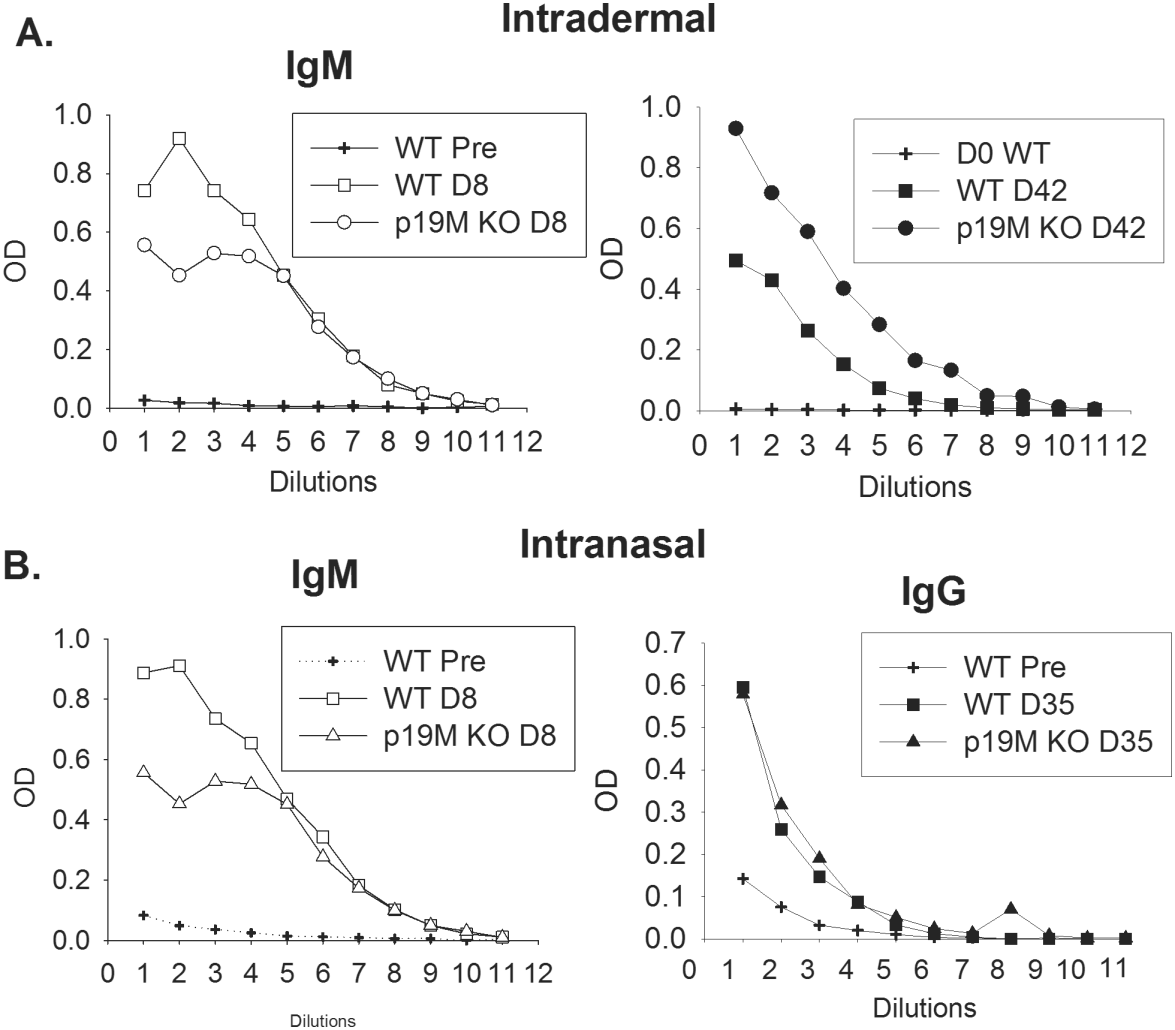
Serum antibodies are comparable between WT and p19 KO mice infected with LVS by the intradermal or intranasal route. Groups of 5 WT or p19M KO mice were infected with 10^5^ LVS ID, or were anesthetized and infected with 2×10^3^ LVS IN. All mice were bled before infection (pre-bleed; pre), and at the indicated time points during and after infection. Sera from individual mice for each group were pooled and assayed for LVS-specific total IgM and IgG antibodies by ELISA. OD is represented on the Y axis, and two-fold serial dilutions are represented on the X axis, where dilution 1 = 1∶40 dilution. A, left panel) IgM was measured in sera from WT mice or p19M KO mice at day 8 after ID infection. A, right panel) IgG was measured from WT mice or p19M KO mice at day 42 after ID infection. B, left panel) IgM was measured in sera from WT mice or p19M KO mice at day 8 after IN infection. B, right panel) IgG was measured from WT mice or p19M KO mice at day 35 after IN infection. On all panels, results from pre-bleeds from WT mice (WT Pre) are also shown; results from KO mouse pre-bleeds are not shown for clarity, but were the same as those for pre-bleeds from WT mice and essentially negative. Results shown are representative of two experiments of similar design and outcome. Similar results were obtained for sera from p19G mice (data not shown).

To evaluate humoral responses, levels of LVS-specific serum antibodies were measured on day 8 and day 42 in WT, p19M KO, and p19G KO mice following infection with 10^5^ LVS ID. There were no detectable anti-LVS IgM and IgG antibodies in the pre-vaccination sera (titer <40, and titration curves for the pre-bleed sera from WT mice are shown in Fig. 3A; data not shown for other groups). At day 8, the IgM anti-LVS titer for sera from LVS-infected WT mice was 10240, compared to 20480 for the p19M KO mice (Fig. 3A). IgG anti-LVS titers were low in all groups at this early time point. The IgG titers at day 42 were also similar for WT and p19 KO mice, such that anti-LVS titers for WT mice were 2560 and p19M KO mice were 10240. The results obtained for the IgG subclasses in sera obtained on day 42 after LVS infection are described in Table 1, where the levels for each subclass were also roughly equivalent amongst the different groups of mice. The serum antibody titers were also measured following IN LVS infection with 2×10^3^ CFU. Pre-bleed serum had low levels of IgM and IgG as expected (titration curves for sera from WT mice are shown in Fig. 3B; data not shown for the other groups). At day 8 after IN LVS infection, the IgM titer was 1280 for sera from the LVS-infected WT mice and 640 for the p19M KO mice (Fig. 3B). By day 35 after IN LVS infection, sera from LVS-infected WT mice and p19M KO mice had the same IgG titer of 2560 (Fig. 3B). Evaluation of the IgG subclass profiles also revealed few differences between sera from the LVS-infected WT mice and p19M KO mice (Table 1), and similar results were found using p19G KO mice (data not shown). Collectively, therefore, although some minor differences in dilution curves and titers were found, overall serum titers for IgM, total IgG, and the IgG subclasses were similar between WT and IL-23p19 KO mice for both infection types.

**Table 1:** Similar titers of serum antibodies titers in LVS-vaccinated WT and IL-23 KO mice.

	WT	IL-23 KO	WT	IL-23 KO
	Intradermal (Day 42)^a^		Intranasal (Day 35)	
**Isotype**				
IgM	40960	10240	1280	640
Total IgG	1280	10240	2560	2560
IgG_1_	640	5120	40	40
IgG_2b_	640	5120	640	2560
IgG_2c_	320	2560	80	640
IgG_3_	160	160	320	320

a Groups of 5 WT or IL-23 KO mice (p19M KO and p19G KO) were infected with 10^5^ LVS ID and sera collected at Day 42, or were anesthetized and infected with 2×10^3^ LVS IN and sera collected at day 35. Sera from individual mice for each group were pooled and assayed for LVS specific total IgM and IgG antibodies, as well as the IgG subclasses (IgG_1_, IgG_2b_, IgG_2c_, and IgG_3_) by ELISA. Serum titers were calculated as described in Materials and Methods. Results shown are representative of two experiments of similar design and outcome.

### Characterization of the role of IL-23p19 during secondary immune responses to LVS infection

We next tested the ability of vaccinated WT and IL-23 KO mice to survive maximal large doses of secondary lethal LVS challenge. Initial experiments demonstrated that both LVS-immune WT mice and KO mice survived at least 5×10^5^ CFU LVS IP (5000 LD_50_s). Therefore final experiments used maximum feasible doses. Groups of 4 to 10 WT, p19M KO, or p19G KO mice were vaccinated with LVS ID or IN. Mice were challenged 42 days later with either 2×10^6^ LVS IP or 8×10^7^ LVS IN. When naïve control mice were challenged either with the high IP or IN dose, all succumbed to challenge as expected (Table 2A). At this maximal challenge dose, some but not all primed WT mice survived secondary challenge by either route, regardless of the route of initial priming (Table 2A). A similar proportion of IL-23KO mice primed with LVS administered either ID or IN succumbed to this very high IP challenge. In another experiment, there were minimal differences between the survival of IN primed, IP challenged WT and p19G KO mice (Table 2B). Further, p19M KO mice that were primed IN and challenged with a lower 5.7×10^5^ IP LVS dose all survived (data not shown). When mice were primed with LVS ID or IN and challenged with the highest available IN dose, the majority of mice from all groups survived (Table 2A). In all combinations, the range of times to death were similar between LVS-infected WT mice and p19 KO mice (Table 2). In sum, while there may be a slightly increased susceptibility to high dose IP challenge after vaccination for LVS-immune IL-23 KO mice compared to WT mice, any difference is subtle. LVS-immune WT and KO mice survive high dose IN LVS challenge equally well.

**Table 2:** Similar survival of lethal challenge by LVS-vaccinated WT and IL-23 KO mice.

	Strain	Primary Infection	SecondaryChallenge	Deaths/Total	Average TTD(Range of Days)
Exp. 1^a^	C57BL/6	PBS	1.7×10^6^ IP	4/4	5 (N/A)
	C57BL/6	2×10^3^ IN	1.7×10^6^ IP	2/3	5 (N/A)
	p19G KO	2×10^3^ IN	1.7×10^6^ IP	2/4	5 (4–6)
	p19G KO	2×10^3^ IN	1.7×10^6^ IP	0/3	-
Exp. 2^b^	C57BL/6	PBS	2×10^6^ IP	3/3	5 (N/A)
	C57BL/6	10^5^ ID	2×10^6^ IP	1/5	7 (N/A)
	p19G KO	10^5^ ID	2×10^6^ IP	4/5	6 (4–9)
	p19M KO	10^5^ ID	2×10^6^ IP	4/4	5 (3–6)
	C57BL/6	10^2^ IN	2×10^6^ IP	2/5	6 (N/A)
	p19G KO	10^2^ IN	2×10^6^ IP	4/5	4 (N/A)
	C57BL/6	PBS	8×10^7^ IN	5/5	6 (N/A)
	C57BL/6	10^6^ ID	8×10^7^ IN	0/5	-
	p19G KO	10^6^ ID	8×10^7^ IN	0/5	-
	p19M KO	10^6^ ID	8×10^7^ IN	0/5	-
	C57BL/6	10^2^ IN	8×10^7^ IN	0/10	-
	p19G KO	10^2^ IN	8×10^7^ IN	2/8	12 (6–18)

a In experiment 1 (Exp. 1), WT C57BL/6J mice or IL-23 KO mice (either p19M KO or p19G KO mice, as indicated) were infected either with 10^5^–10^6^ ID or 10^2^ IN LVS. One month later, when primary infection was cleared, surviving mice were given a secondary high dose challenge of 2×10^6^ IP or 8×10^7^ LVS IN. Naïve control mice treated with PBS ID or IN were challenged as indicated IN or IP, where all mice died.

b Exp. 2, mice were given a primary vaccination of 2×10^2^ or 2×10^3^ IN and challenged with 2×10^6^ IP. The average time to death (TTD) of those mice that died within a group is also shown, as well as the range of the days of death. Groups where all the mice succumbed on the same day are given a range of (N/A). Data are representative of three experiments of similar design and outcome.

**Figure 4:**
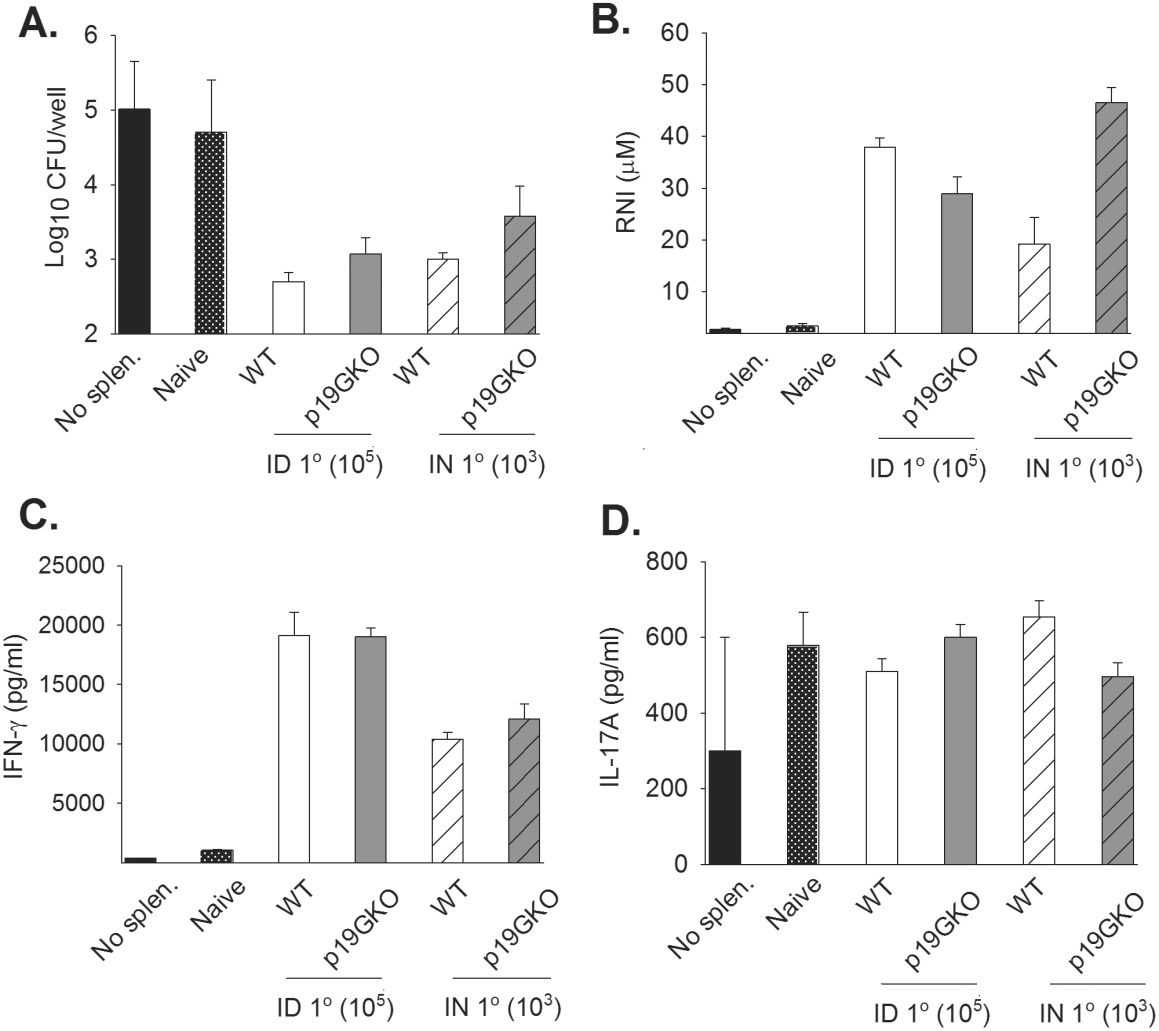
T cell functions and cytokine production are similar between WT mice and p19G KO mice, regardless of priming route. WT mice and p19G KO mice were infected with 10^5^ LVS ID or 10^3^ LVS IN. Mice were sacrificed after 8 weeks and splenocytes isolated; splenocytes were also obtained from naïve mice. Bone marrow-derived macrophages from WT mice (shown) or p19G KO mice (not shown) were infected with LVS, and then 5×10^6^ homologous splenocytes were added to triplicate wells of infected macrophages. A) Intracellular bacterial burdens were assessed immediately after infection at 0 hours (not shown) and at 72 hours after infection. B) Supernatants were harvested from cultures at 72 hours after infection, and the levels of RNI measured. Supernatants were also assayed for cytokines by sandwich ELISA, including C) IFN-γ and D) IL-17A. IL-12p40 and IL-12p70 were also measured (data not shown). Results are shown for one representative experiment of three experiments of similar design and outcome. *p value≤0.05 by Student’s *t* test.

We next investigated the role of IL-23 in acquired memory T cell responses to LVS. To do this, we employed an *in vitro* co-culture system that quantitates the ability of LVS-primed splenocytes, dominated by T cells, to control intramacrophage LVS replication. WT mice and p19M KO mice were vaccinated with either 10^5^ LVS ID or 10^3^ LVS IN. Eight weeks later, splenocytes from vaccinated mice as well as from naïve mice were prepared. Bone marrow-derived macrophages from WT mice (Fig. 4A) and p19G KO mice (data not shown) were infected with LVS, and naïve or immune splenocytes added to the cultures. As expected, LVS replicated exponentially in macrophages with no splenocytes, or in those with naïve splenocytes (Fig. 4A). The addition of splenocytes from WT mice and p19G KO mice, primed with LVS by either ID or IN routes, controlled intracellular LVS replication to a comparable degree (Fig. 4A). Further experiments in which the numbers of splenocytes were reduced 10 and 100-fold also revealed no differences in relative control between LVS-immune WT and KO cells (data not shown). Finally, no differences were observed between WT and p19G KO or p19M KO splenocytes harvested four months after priming (data not shown). Thus this function of LVS-immune T cells is comparable between WT mice and p19 KO mice.

To evaluate memory T cell functions further, culture supernatants were harvested and the levels of reactive nitrogen intermediates (RNI) and cytokines measured. RNI levels were equivalent among the different splenocyte groups (Fig. 4B). In the experiment shown, there was a significant increase in RNI in the p19G KO samples from IN primed splenocytes compared to WT samples, but this result was not consistently seen in repeated experiments. The levels of IFN-γ and IL-17A were also similar across supernatants from the primed groups (Fig. 4C and Fig. 4D). Finally, there were no differences in amounts of IL-12p40, IL-12p70, or TNF-αin supernatants from either type of culture (data not shown).

Taken together, therefore, our data suggest that IL-23p19 is largely dispensable for both primary (innate) and secondary (acquired) immunity to LVS.

## Discussion

Unlike IL-12p35 KO mice, IL-12p40 KO mice infected with LVS either ID or IN exhibit a chronic LVS infection that is never cleared from the animals, and have altered antibody responses [13] (Elkins *et al*., unpublished data). These observations suggested a role for IL-23, which is comprised of p40 and p19 subunits. However, we found that p19, and thus IL-23, is largely dispensable for either primary or secondary resistance to LVS infection. The LD_50_s for IN and ID LVS infection; bacterial burden in organs; serum antibody titers; immune T cell function, including cytokine production; and ability to resist secondary challenge are all equivalent between IL-23 KO and WT mice. Of note, here we employed two independently-derived types of p19 KO mice, and found essentially identical results with each. Therefore, the role that IL-12p40 plays in host resistance to LVS must be independent of its participation in the IL-23 heterodimer. IL-12p40 can form a biologically active homodimer [15], and perhaps it is this molecule, or p40 monomer, that is important for clearance of LVS. Alternatively, there may be another as-yet undiscovered role for IL-12 p40.

IL-23 is important for immunity to other pathogens, including intracellular bacteria. IL-23p19 KO mice are more sensitive to infection with *Listeria monocytogenes*, exhibiting increased liver bacterial burdens and increased mortality [19]. The loss of IL-23 resulted in decreased IL-17 production, another integral component of the immune response. IL-23p19 KO mice infected with *Klebsiella pneumoniae* also have increased mortality and decreased cytokine and chemokines responses in lungs, including IL-17 and IL-6 [20]. The link between IL-23 and resistance to *Toxoplasma* infection is more tenuous. IL-12p40 KO mice are more sensitive to *T. gondii* infection, and giving these infected mice recombinant IL-23 improves survival [32]. However, there is no increased susceptibility in IL-23 p19 KO mice, which in one case had improved survival; further, there were no changes in T cell populations or cytokine production in response to *T. gondii* infection [32], [33]. When infected by aerosol with *M. tuberculosis*, IL-23p19 KO mice have slightly increased bacterial burdens in the spleen at a late (∼day 150) time point but not at early time points [18]. These studies further demonstrate that the phenotype of IL-23 p19 KO mice can often be different from that seen in IL-12 p40 KO mice and p35/p19 double KO mice, suggesting that IL-23 has non-overlapping functions with IL-12p70 but in some circumstances can partially compensate for the loss of IL-12p70.

IL-23 is also an important component of humoral immunity as well as the delayed type hypersensitivity (DTH) response, a strongly T-cell dependent pathway [25]. IL-23p19 KO mice are unable to mount a full DTH response. Further, they have impaired antigen-specific antibody production, although they exhibit normal T-independent B cell responses. The deficiencies seen in IL-23p19 KO mice are due at least in part to a defect in memory T cell activation. Here, however, we did not observe any differences in memory T cell function, as reflected in the ability of LVS-immune T cells to control the intramacrophage replication of LVS (Fig. 4A) or to produce Th1-related cytokines (Fig. 4C, and data not shown). Further, anti-LVS serum antibodies were comparable between WT mice and p19 KO mice, with only minor differences of uncertain biological significance noted (Fig. 3, Table 1).

IL-23 is also important during autoimmune responses, and p19 KO mice are resistant to experimental autoimmune encephalomyelitis (EAE; reviewed in [34]). IL-23 promotes autoimmunity by inducing the expansion of IL-17-producing Th17 cells, which in turn cross into the central nervous system and promote EAE pathology [35]. Th17 cytokines have been implicated in several other autoimmunity models, including multiple sclerosis, arthritis, inflammatory bowel disease, psoriasis, and lupus [36]. T helper type 17 (Th17) cells are distinct from Th1 and Th2 subsets, and are characterized as being highly proinflammatory and by their unique pattern of cytokine expression. The combination of TGF-β and IL-6 or IL-21 is required to triggering the Th17 differentiation pathway, thus inducing IL-17 secretion in naïve T cells [37]. However, IL-23 is also an important contributor to the promotion of a productive and sustained Th17 response [37].

IL-17 has been implicated in resistance a number of pathogens including *Mycobacterium tuberculosis*, *M. bovis* BCG, *Salmonella enterica*, *Listeria monocytogenes*, *Klebsiella pneumoniae*, and *Salmonella typhimurium* (reviewed by [38]). Similarly, there is evidence for an important if subtle role for IL-17 in immunity to LVS. Cowley *et al.* described a major CD4^−^CD8^−^ (DN) T cell subset that expands during pulmonary LVS infection and produces large quantities of IFN-γ and IL-17A [39]. The recruitment of these IL-17A^+^ DN T cells was limited to the lungs in IN-infected mice, as they were not found in the spleens of these mice, or in the lungs or spleens of ID-infected animals. In concordance with this, IL-17A KO mice have increased bacterial burdens compared to WT mice following pulmonary LVS infection, but not during parenteral infection. Further, DN T cells protected mice from LVS in the absence of all other T cell populations, and this protection is IL-17A-dependent. In other studies, mice infected IN with LVS and given exogenous IL-17A by the same IN route exhibited slightly increased survival times [40]. The same was true when infected mice were given IL-23, by either IP or IN route. Conversely, treating mice with anti-IL-17A antibody during infection resulted in decreased survival. Here, however, we saw no clear dependence on IL-23 for survival of primary IN infection (Fig. 1), and no obvious differences in amounts of IL-17 produced by LVS-immune T cells during co-culture with LVS-infected macrophages (Fig. 4D).

Given the importance of IL-17/Th17 immunity and the link to IL-23, we also measured the levels of IL-17 organ homogenates during the entire course of LVS infection in WT mice and IL-23 KO mice. However, we did not see any detectable differences in IL-17 production in the lungs, livers, or spleens during either primary ID or IN LVS infection (data not shown). Therefore, an intact IL-17 response in the absence of IL-23 may be contributing to host resistance. However, we did not specifically analyze the Th17 population in the IL-23 KO mice; thus there may still be some alteration in the Th17 response, although if so such a difference does not grossly impact resistance to LVS.

In the course of investigating the role of IL-17, Lin *et al.* [41] found IL-23 KO mice to be more susceptible than WT mice following administration of a single dose intratracheal LVS infection. This is in disagreement with our findings that IL-23 is dispensable. However, we used IN and ID delivery versus an intratracheal route, which may lead to different outcomes. We also note, however, that Lin *et al.* reported unusually high organ burdens during LVS infections in all WT mice and knockout mice compared to those seen by us and by many other investigators. These unusual observations raise the possibility that technical issues may be a major contributor to the disparate results.

Taken together, we conclude that IL-23 is largely dispensable for host resistance to LVS, and that the IL-17/Th17 pathway is likely still intact and activated independently of IL-23. The mechanism by which IL-12 p40 promotes clearance of LVS remains unknown, but these data rule out its participation in the IL-23 heterodimer as one such mechanism. The obvious candidate is p40 itself, either as a monomer or as a homodimer. Although p40 is made in abundance after LVS infection and after infection with other intracellular bacteria such as *M. tuberculosis*, to date only limited biological roles for p40, independently of IL-12, have been described. In vitro studies suggest that p40 can serve as a receptor decoy, blocking recognition of IL-12 p70 by the IL-12 receptor [7]; this effect may contribute to increased susceptibility to diseases such as leishmaniasis [42]. However, p40 also has a role as an agonist, especially as a homodimer. In vitro, p40_2_ stimulates production of lymphotoxin-α and IL-16 by macrophages, microglia, and other cells [43], [44], and reduces development of FoxP3^+^ regulatory T cells by inducing production of nitric oxide [45]. In vivo, absence of p40 results in increased susceptibility to mycobacterial infection compared to the absence of p35 and IL-12 [46], and p40_2_ stimulates macrophage and dendritic cell migration during mycobacterial infection [47], [48]. Current studies in our laboratory are therefore directly investigating the role for IL-12p40 itself.
